# *EXO1* Plays a Carcinogenic Role in Hepatocellular Carcinoma and is related to the regulation of *FOXP3*

**DOI:** 10.7150/jca.40673

**Published:** 2020-06-16

**Authors:** Guang Yang, Keshuai Dong, Zunyi Zhang, Erlei Zhang, Binyong Liang, Xiaoping Chen, Zhiyong Huang

**Affiliations:** 1 Hepatic Surgery Center, Tongji Hospital, Tongji Medical College, Huazhong University of Science and Technology, Wuhan, China.; 2 Department of Hepatobiliary and Laparoscopic Surgery, Renmin Hospital, Wuhan University, Hubei Key Laboratory of Digestive System Disease, Wuhan, China.

**Keywords:** *EXO1*, * FOXP3*, Hepatocellular carcinoma, Metastasis, Transcriptional regulation

## Abstract

Exonuclease 1 (*EXO1*), a member of the RAD2 nuclease family, was first described as possessing 5' to 3' nuclease activity and 5' structure-specific endonuclease activity. Here, we show that *EXO1* is significantly upregulated in HCC tumor tissues and that high *EXO1* expression is significantly correlated with liver cirrhosis. We further demonstrate that *EXO1* knockdown decreases proliferation and colony forming abilities of HCC cells *in vitro* and tumorigenicity *in vivo*, as well as decreases migration and invasive capabilities of HCC cells. Alternatively, *EXO1* overexpression significantly increases the proliferation, colony forming ability, and migration and invasive capabilities of HCC cells *in vitro*. Additionally, we truncated a region upstream of the transcription start site (TSS) of *EXO1* and used the region with the strongest transcriptional activity to predict that the transcription factor *FOXP3* can bind to the *EXO1* promoter. Bioinformatics analysis found that *FOXP3* was positively correlated with *EXO1* and luciferase reporter assays and RT-PCR confirmed that *FOXP3* could enhance the transcriptional activity of *EXO1*. CCK-8 assays showed that depletion of *FOXP3* further reduces cell proliferation ability after knocking down of *EXO1 in vitro*. Taken together, our findings indicate that *EXO1* acts as an oncogene in HCC and its expression level is related to *FOXP3* activity.

## Introduction

Hepatocellular carcinoma (HCC) is the fifth most common cancer and the third leading cause of cancer-related deaths worldwide 1. Most patients are initially diagnosed at an advanced stage in the disease and thus, the curative effect of hepatectomy is often unsatisfactory. The 5-year recurrence rate of HCC after hepatectomy exceeds 80%, and the 5-year survival rate is only 30%-70% 2, 3. Therefore, in-depth study of the molecular mechanisms involved in the development of HCC is of great significance to develop effective therapeutic drugs and prevent the recurrence of HCC after hepatectomy.

Exonuclease 1 (*EXO1*) is a member of the *Rad2/XPG* family of nucleases and was originally identified in *Schizosaccharomyces pombe* 4. *EXO1* possess 5' to 3' exonuclease activity and structure-specific endonuclease activity and plays an extremely important role in biological processes such as DNA replication, DNA mismatch repair (MMR), DNA double-strand break repair (DSB) and telomere maintenance 5-9. Deletion of the *EXO1* gene causes genomic instability and leads to impaired DNA damage repair and meiosis defects 7, 10-13. However, studies have shown that abnormally high expression of *EXO1* is related to the occurrence, development and prognosis of various malignant tumors. Kretschmer et al. reported for the first time that *EXO1* is significantly elevated in breast ductal carcinoma and invasive ductal carcinoma 14. A recent study by de Sousa et al. has shown that *EXO1* expression is significantly elevated in glioma, and high *EXO1* expression is an independent risk factor for poor prognosis of patients 15. Dai et al. reported that *EXO1* is upregulated in HCC specimens and *EXO1* overexpression may also be associated with poor prognosis in HCC patients 16. In addition, several single nucleotide polymorphisms (SNPs) in the *EXO1* gene are associated with tumor susceptibility in a variety of tumors including liver, gastric, ovarian, cervical, breast, and colorectal cancers as well as head and neck squamous cell carcinoma 17-25. Among them, rs1047840 (K589E), a common SNP genotype, increases the risk for developing non-viral liver cancer, colorectal cancer and lung cancer 17, 26, 27. However, the role of *EXO1* in the invasion and metastasis of HCC and the upstream transcriptional regulation of *EXO1* is still poorly understood.

Forkhead box P3 (*FOXP3*) is a member of the forkhead/winged-helix family of transcription factors, which was first identified in T-regulatory (Treg) cells as an essential protein for regulating immune system development and function 28. *FOXP3* is primarily known for its function in regulating the CD4^+^CD25^+^ regulatory cells and for defining their immunoregulatory phenotype 29. Like other transcription factors, *FOXP3* can bind to numerous enzymes and microRNAs to up- or downregulate a large number of genes 30. Dysregulation of *FOXP3* has been reported in the context of various tumors types indicating that it could be a poor prognostic factor in colorectal cancer and bladder cancer 31, 32, but a potential tumor suppressor gene in breast cancer 33, 34. Nevertheless, whether *FOXP3* can regulate the transcription of *EXO1* is poorly understood.

In this study, we discovered that *EXO1* expression is significantly higher in HCC tumor tissues. It was also found that *EXO1* could promote the proliferation of HCC cells *in vitro* and *in vivo*, as well as promote the migration and invasion of HCC cells *in vitro*. *FOXP3* could lead to the upregulation of *EXO1* at the transcriptional level, where it could act as an oncogene in HCC as well. Our research revealed the relationship between the abnormal expression of *EXO1* and malignant biological characteristics of HCC and might lead to the development of novel anti-cancer therapeutics for HCC treatment.

## Materials and Methods

### Gene chip analysis and human HCC samples

99 HCC tumor tissues and matched non-tumor tissues from archived patients at the Hepatic Surgery Centre, Tongji Hospital of Huazhong University of Science and Technology (Wuhan, China) were collected between January 2017 and December 2018. The diagnosis of HCC was confirmed by pathological examination and the differentiation status was graded according to the Edmondson grading system. After surgical resection, samples were stored at -80˚C immediately. Written informed consent was obtained from all subjects, and this study was conducted in accordance with the Declaration of Helsinki and approved by the Ethics Committee of Tongji Hospital, Huazhong University of Science and Technology.

HCC tissues and adjacent non-tumor liver tissues were placed in a cryogenic tank with dry ice and were delivered to the Shanghai Biotechnology Corporation. The Agilent version 16.0 was applied to screen the gene expression profiles of DNA repair pathways between HCC tissues and their adjacent liver tissues.

### Immunohistochemical (IHC) staining assays

Formalin-fixed paraffin-embedded sections were deparaffinized in xylene and rehydrated with an ethanol gradient. The sections were placed in citric acid antigen repair buffer (Wuhan Goodbio Technology Corporation, Wuhan, China) and antigen repair was performed by microwaving on a low setting for 10 min. Endogenous peroxidases were blocked by 3% H_2_O_2_. Sections were then incubated overnight with an anti-*EXO1* antibody at 1:200 (abs115859, Absin, Beijing, China) at 4℃. Secondary antibodies were incubated and peroxidase activity was detected using the EnVision kit (Dako, Glostrup, Denmark). Hematoxylin (Sigma Aldrich, St. Louis, MO, USA) was used for nuclear counterstaining. IHC scores were calculated as the positive staining intensity scores multiplied by the stained positive cells scores. Overall scores of >6 and ≤6 were defined as high expression or low expression of *EXO1*, respectively.

### Cell culture

The HCC cell lines Hep3B, Huh7, SK-Hep1, Bel-7402, and SMMC7721 were purchased from the China Center for Type Culture Collection (Wuhan, China), and HCC cell lines MHCC-97H and HCC-LM3 were obtained from the Liver Cancer Institute, Zhongshan Hospital, Fudan University (Shanghai, China). The HCC cell line HLE was obtained from the Department of Molecular Biology, Peking University (Beijing, China). The HCC cell line PLC, human fetal liver cell line HL-7702, hepatoma cell line HepG2 and lentivirus packaging cell line 293T were purchased from the cell bank of the Chinese Academy of Sciences (Shanghai, China). Cell lines were cultured in Dulbecco's Modified Eagle's medium (DMEM, Hyclone, Utah, USA) containing 10% fetal bovine serum (Gibco, Carlsbad, CA). All cell lines were incubated in a humidified atmosphere with 5% carbon dioxide at 37 °C.

### Generation of EXO1 overexpression and knockdown constructs and FOXP3 knockdown construct

Full-length *EXO1* coding sequence (CDS) was purchased from Vigene Biosciences (Shandong, China). *EXO1* was subcloned into pLenti-CMV-GFP-Puro (plasmid# 17448; Addgene, Cambridge, MA, USA), according to the manufacturer's instructions.

For *EXO1* knockdown assays, three shRNA (sh*EXO1*-TRCN0000039788, sh*EXO1*-TRCN0000039789, and sh*EXO1*-TRCN0000010331) sequences were obtained from the Sigma website (www.sigmaaldrich.com). All these shRNAs were subcloned into the lentiviral vector pLKO.1 puro (plasmid # 8453; Addgene, Cambridge, MA, USA) according to the manufacturer's instructions. Either the sh*EXO1* constructs, *EXO1* overexpression constructs or vector control plasmids were transfected into the 293T cell line by Lipofectamine 2000 (Invitrogen, Thermo Fisher Scientific, MA, USA) using the lentivirus packaging system. The plasmids pMD2.G and psPA×2 were gifts from Didier Trono (plasmids# 12259 and# 12260; Addgene).

The supernatant-containing lentivirus produced by transfected 293T cells was collected 72 hours later. Collected lentiviral supernatants were filtered through a 0.45 μm filter (Millipore, USA). Target cells were infected with filtered lentivirus and 8 μg/mL polybrene (Sigma-Aldrich) to generate stable cell lines; treatment with 8 μg/mL puromycin (Ann Arbor, MI, USA) for 7 days was used to screen the positive cells.

For si*FOXP3* transfection assays, three siRNA (si*FOXP3*-1 stB0001154A, si*FOXP3*-2 stB0001154B and si*FOXP3*-3 stB0001154C) sequences were purchased from RiboBio (Guangzhou, China). About 3×10^5^ HLF or Huh7 cells/well were seeded into a 6-well plate and infected with 5 μL of siRNAs after adherence. Lipofectamine 3000 (Invitrogen) was used for transfection.

### Western blot analysis

Total protein was extracted from the cells using RIPA lysis buffer. Protein concentrations were measured with a BCA Protein Assay Kit (Thermo Fisher Scientific, MA, USA). Protein lysates were separated by SDS-PAGE and transferred onto a polyvinylidene difluoride (PVDF) membrane (EMD Millipore, Billerica, MA, USA). The membrane was blocked in non-fat milk in Tris-buffered saline containing 0.1% Tween-20 for 2 h and incubated with primary antibody (*EXO1*, ab95012, Abcam, Cambridge, MA, USA; β-actin, Cell Signaling Technology, Danvers, MA, USA) at 4 °C overnight. Then, membranes were incubated with secondary antibodies and signals were detected by Bio-rad chemiluminescence system.

### Cell proliferation assay

Cells were inoculated into 96-well plates with 1×10^3^ cells/well, and 100 μL of 10% cell counting kit-8 (CCK-8, Dojindo, Kumamoto, Japan) solution was added into each well at 0, 1, 2, 3, 4, and 5 days after cell adherence. The optical density (OD) values were measured by an EXL-800 absorbance reader at a wavelength of 450 nm at the indicated time points (after Cell Counting Kit-8 solution was added to the wells for 2 hours).

### Cell migration and matrigel invasion assays

Cell migration and invasion assays were performed using transwell chambers (Corning, USA) with or without Matrigel (BD Biosciences, CA, USA). About 1.5×10^4^-10×10^4^ cells in medium without FBS were seeded on transwell chambers with or without Matrigel. Below the transwell chambers, DMEM containing 10% fetal bovine serum (FBS) was added. An automated cell counter (Nexcelom Bioscience, USA) was used to quantify the number of migratory or invasive cells. Six fields at a magnification of ×200 were randomly selected for counting stained cells.

### Cell colony formation

*EXO1*-knockdown or -overexpressing HCC cells (HLF, Huh7, Bel7402) were plated in 6-well plates (1000 cells/well) and culture media was replaced every 3 days. After 2-3 weeks when clones were visible to the naked eye, cells were fixed with 4% polyformaldehyde for 15 minutes and stained with crystal violet (Sigma-Aldrich Corporation) for 15 minutes. Photographs of the 6-well plates were taken, and the differences were counted.

### Xenograft tumorigenicity assays

Animal studies were approved by the Ethics Committee of Tongji Hospital, Huazhong University of Science and Technology. All animal experiments were performed under the guidelines of the Interdisciplinary Principles and Guidelines for the Use of Animals in Research, Testing, and Education by the New York Academy of Sciences, Ad Hoc Animal Research Committee. 5-week-old male BALB/c (nu/nu) mice were obtained from the HFK BioScience Corporation (Beijing, China). All the mice were bred under specific pathogen-free (SPF) conditions at the Laboratory Animal Center of the Tongji Hospital. For subcutaneous tumorigenesis assays, HLF vector- and HLF sh*EXO1*-788-transfected cells (2×10^6^) were suspended in 100 μL of Dulbecco's modified Eagle's medium (DMEM) and injected subcutaneously into the left and right back of nude mice, respectively. Nude mice were monitored after the injection and sacrificed at day 30 after cell inoculation. The final tumor volumes were calculated according to the equation: V (volume, mm^3^) = 0.5 × L (length, mm) × W^2^ (width, mm^2^).

### RNA extraction and quantitative real-time PCR

Total RNA was extracted using TRIzol Reagent (Takara, Dalian, China) and reverse transcription was performed using a reverse-transcription PCR kit (TIANGEN, Beijing, China) according to the manufacturer's instructions. Quantitative real-time PCR was carried out using SYBR Green PCR master mix (TIANGEN, Beijing, China) on a Bio-Rad CFX system (Bio-Rad, Hercules, CA, USA) with the following primers:

*EXO1*-forward: 5' - TGAGGAAGTATAAAGGGCAGGT -3';

*EXO1*-reverse: 5'- AGTTTTTCAGCACAAGCAATAGC - 3'.

*FOXP3*-forward: 5'- GTGGCCCGGATGTGAGAAG - 3';

*FOXP3*- reverse: 5'- GGAGCCCTTGTCGGATGATG - 3'.

*GAPDH*- forward: 5'- GGAGCGAGATCCCTCCAAAAT- 3'.

*GAPDH*- reverse: 5'- GGCTGTTGTCATACTTCTCATGG- 3'.

The Ct values of *EXO1* were equilibrated to those of the internal control *GAPDH*.

### Luciferase reporter assay

The region 2000 bp upstream of the *EXO1* translational start site was amplified from the genomic DNA of the Huh7 cell line by PCR. *EXO1* promoter regions of different lengths were subcloned into pGL4.17 (Promega, Madison, WI, USA). The open reading frame of *FOXP3* and other candidate genes was amplified by PCR and then cloned into pcDNA 3.1(+) (Invitrogen, Thermo Fisher Scientific, MA, USA) to produce a series of products such as pcDNA 3.1-*FOXP3* and so on. pGL4.17 and pcDNA3.1 were used as controls. pRL-TK was purchased from Promega (Madison, WI, USA). About 1 × 10^5^ Huh7 cells/well were seeded into a 24-well plate and co-transfected with 4 ng of the pRL-TK, 200 ng of pgl4.17 and 400 ng of pcDNA3.1 after adherence. Lipofectamine 2000 (Invitrogen) was used for transfection. Cell lysates were prepared using passive lysis buffer (Promega) 48 h after transfection. A luciferase activity was performed using the Dual-Luciferase Reporter Assay System (Promega, Madison, WI, USA) according to the manufacturer's instructions. The relative luciferase activity was measured using a GloMax 20/20 Luminometer (Promega).

### Data acquisition and processing

Data for bioinformatics analysis were collected from The Cancer Genome Atlas (TCGA), Oncomine and Gene Expression Omnibus (GEO) databases. The cor.test () function in the R (version 3.5.2; https:// www.r-project.org/)) was used to calculate and verify the correlation coefficients between the *EXO1* and *FOXP3*. Then R packages ggplot2 (version 3.1.1) was used for plotting.

### Statistical analysis

Statistical analyses were performed with IBM SPSS Statistics 21.0 (SPSS, Chicago, IL, USA) or Prism 6.0 (GraphPad Software, La Jolla, CA) software. The results were presented as the mean ± standard deviation (SD) or mean ± standard error of mean (SEM). Quantitative data were compared by a two-tailed Student's t-test, One-way ANOVA and Mann-Whitney U test when applicable. Categorical data were analyzed by the χ^2^ test or Fisher's exact test. Kaplan-Meier survival analysis (log-rank test) was used to compare HCC patient survival. A two-sided value of *p*<0.05 was considered statistically significant.

## Results

### *EXO1* is an upregulated DNA damage repair gene in HCC

**Figure 1:**
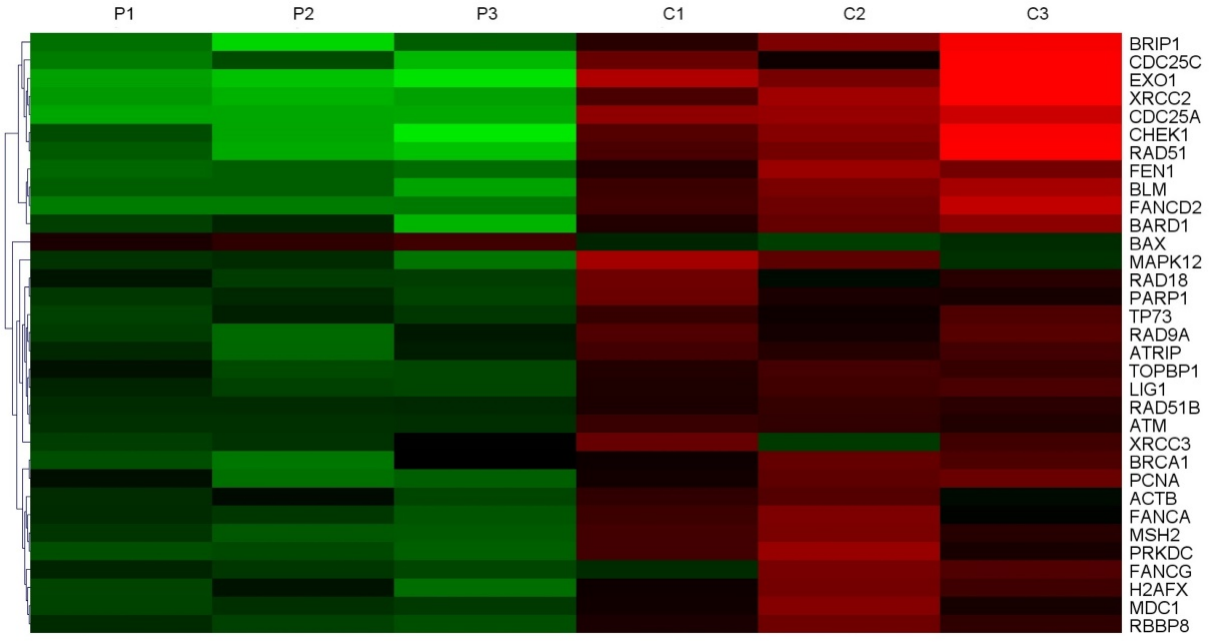
*EXO1* is an up-regulated DNA damage repair gene in HCC. Heat Map of top-ranked upregulated DNA damage repair genes in HCC tissues compared with adjacent normal liver tissues detected by gene chip technology. The results showed that the expression level of 33 molecules in HCC tissues was higher than that in corresponding non-tumor tissues. (P: Pericancerous liver tissue; C: hepatocellular carcinoma tissue).

In order to screen the expression of DNA damage repair genes, three samples of HCC tissues and adjacent non-tumor tissues were collected for whole-genome sequencing. Gene chip analysis showed that 33 DNA damage repair genes were expressed at higher levels in tumor tissues compared to their corresponding non-tumor tissues (Figure 1), and in tumor tissues, 8 of them were expressed at levels greater than 10 times the normal levels. The expression of *EXO1* in HCC tumor tissues was 27.2 times higher than that in peri-cancerous liver tissues (Table 1). Hence, we chose to perform follow-up studies on *EXO1* and focused on its functional roles and mechanisms *EXO1* in driving HCC development and metastasis.

### Clinical significance of *EXO1* in HCC patients

**Figure 2:**
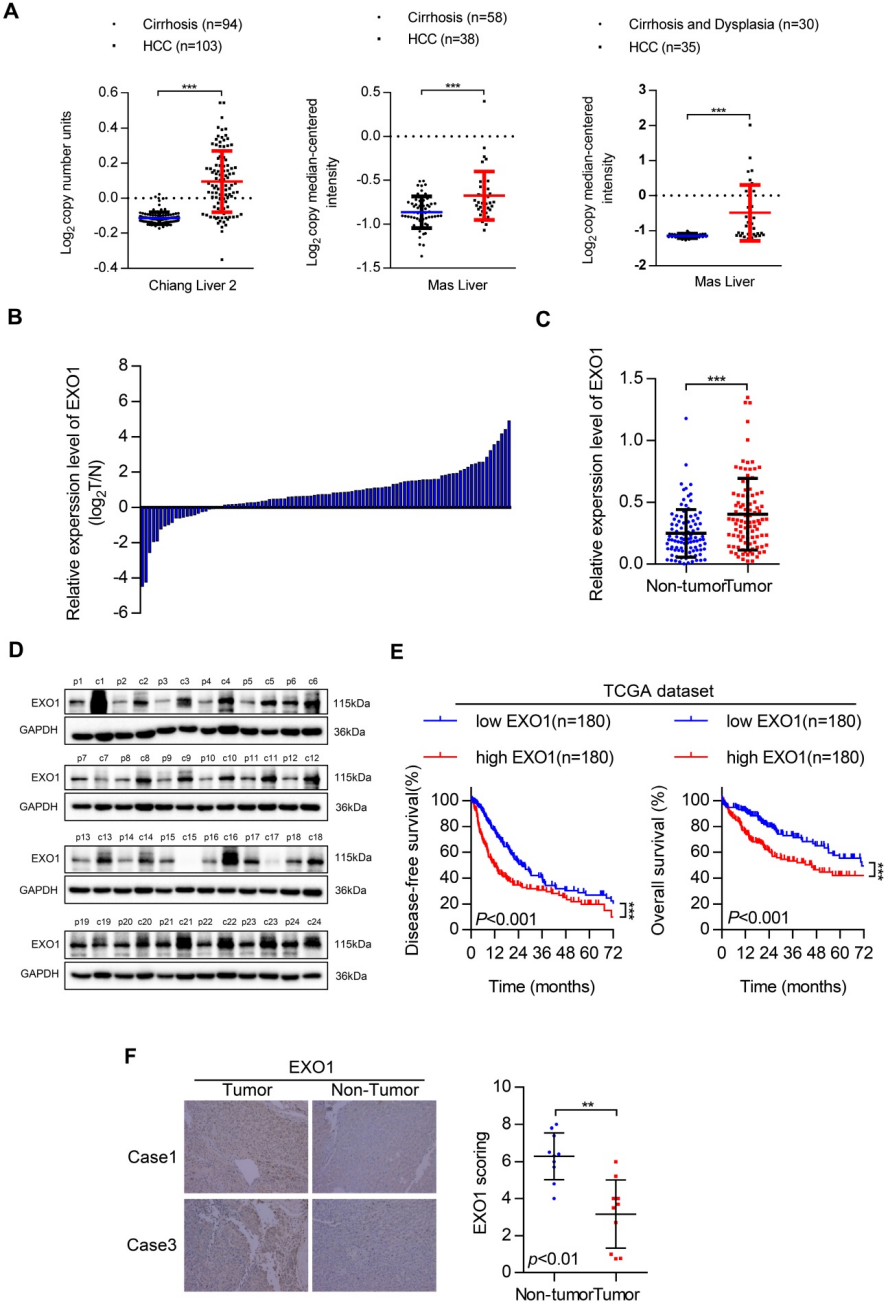
*EXO1* expression is upregulated in HCC and associated with poor outcomes in HCC patients. (A) Dot chart of analysis of *EXO1* expression in HCC tumor tissues and non-tumor tissues in the Oncomine database. (B) The expression of *EXO1* in 99 paired HCC tissues and adjacent non-tumor tissues was detected by western blotting. The bar chart shows the expression level of *EXO1* in HCC tissues, which were quantified and normalized to the corresponding *EXO1* levels in the adjacent non-tumor tissues. (C) Dot chart of *EXO1* expression in the 99 HCC samples. (D) Representative images of Western blots of *EXO1* in 99 HCC tumor tissues and their adjacent non-tumor tissues. (E) Kaplan-Meier analysis showed the disease-free survival curve and overall survival curve of two different groups: patients with high *EXO1* expression and patients with low *EXO1* expression. The data comes from TCGA database. (F) IHC analysis of *EXO1* expression in 10 paired HCC tissues. Results of case 1 and case 3 are used as representative images in the left panels and statistical analysis of *EXO1* expression in the HCC samples in the right panel. (20×, magnification) (*p<0.05; **p<0.01; *** *p*<0.001).

First, we investigated *EXO1* expression in tumor and non-tumor liver tissues in three different clinical studies from the Oncomine database (Figure 2A). The results showed that the expression of *EXO1* was significantly higher in tumor tissues than that in their adjacent non-tumor tissues. Next, the expression level of *EXO1* was detected in 99 HCC samples from Tongji Hospital (Wuhan, China) by western blotting (Figure 2C, D). These results showed that the expression of *EXO1* in HCC tumor tissues was higher (79%) than that in adjacent non-tumor tissues (21%) (Figure 2B). We further explored the relationship between clinicopathological features and the expression level of *EXO1* in HCC patients. Ninety-nine HCC patients were divided into an *EXO1* low-expression group or *EXO1* high-expression group. The detailed information is summarized in Table 2. High expression of *EXO1* was significantly associated with the presence of liver cirrhosis (*p=*0.005). However, the expression of *EXO1* was not correlated with gender, age, or AFP level. We then used the TCGA database to analyze the relationship between *EXO1* expression and long-term prognosis of HCC. Kaplan-Meier analysis revealed that the disease-free survival rate and overall survival rate of patients with high *EXO1* expression were significantly lower than that of patients with low *EXO1* expression (Figure 2E). Subsequently, the expression level of *EXO1* was assessed by immunohistochemistry (IHC) in 10 pairs of tumor tissues and their adjacent non-tumor tissues. As shown in Figure 2F, *EXO1* in hepatocytes was localized to the cytoplasm, and the average IHC score of *EXO1* in tumor tissues was significantly higher than that in adjacent non-tumor tissues. Taken together, these results indicate that *EXO1* expression is significantly upregulated in HCC tissues and high expression of *EXO1* plays an important role in the prognosis of HCC patients and may contribute to the progression of HCC.

### *EXO1* enhances proliferation and colony formation of HCC cells* in vitro*, as well as tumor growth *in vivo*

**Figure 3:**
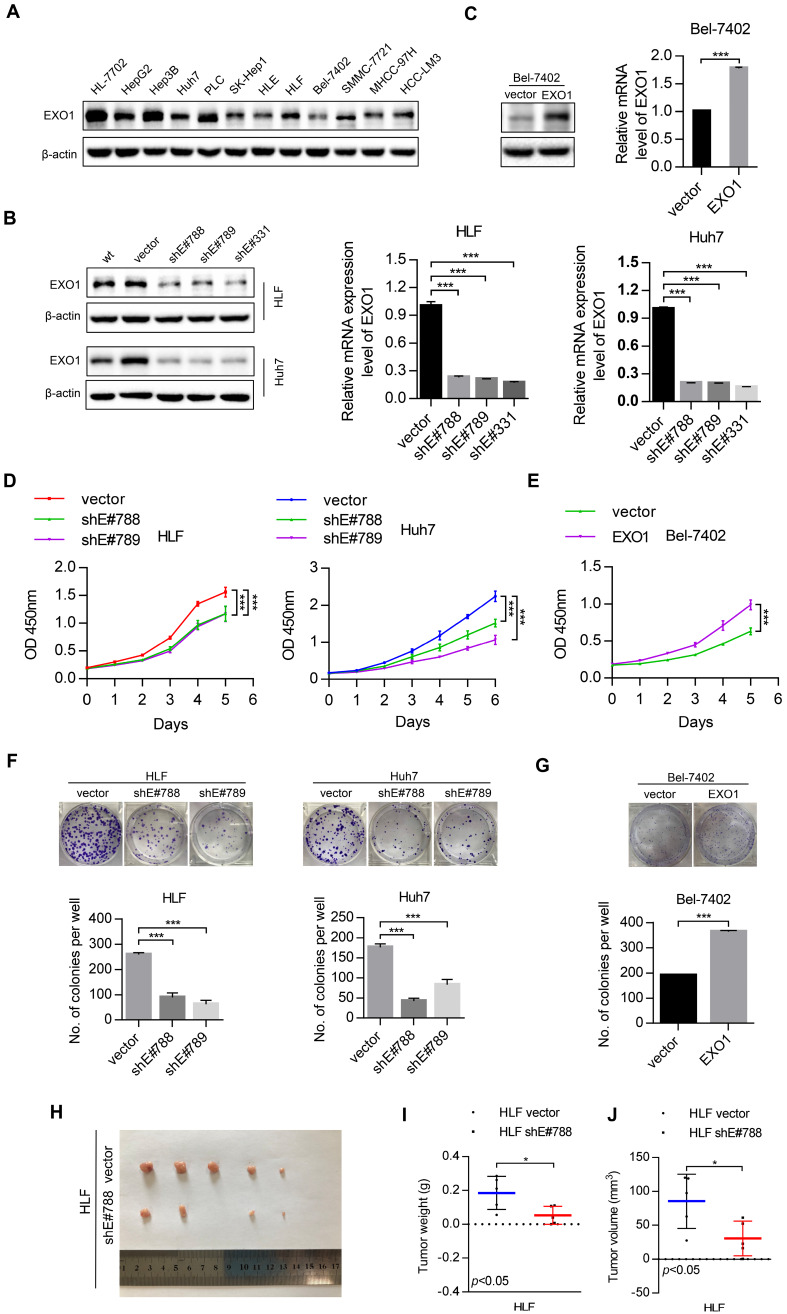
*EXO1* promotes cell proliferation and colony formation in HCC cell lines and proliferation *in vivo*. (A) The expression levels of *EXO1* in one healthy liver cell line, ten HCC cell lines and one HCC lung metastasis cell line were detected by western blotting. (B), (C) Western blotting confirmed that *EXO1* expression was effectively repressed by shRNA in HLF and Huh7 cell lines and *EXO1* was overexpressed in the Bel-7402 cell line. The bar chart shows that *EXO1* mRNA levels were significantly decreased or increased in the corresponding cell lines compared to the vector group. The data are shown as the mean ± SEM. (D), (E) CCK-8 assays indicated that knockdown of *EXO1* significantly reduced cell proliferation abilities while overexpression of *EXO1* increased cell proliferation abilities in the corresponding cell lines. The data are shown as the mean ± SD (n=6) (F), (G) Representative image of colony formation assays of *EXO1* knockdown and *EXO1* overexpression in the corresponding cell lines. The bar chart shows that *EXO1* knockdown reduced colony formation abilities but overexpression of *EXO1* enhanced colony formation abilities compared to their vector groups. Data are represented as the mean ± SD (n=3). (H) Representative image of subcutaneous tumors (n=5). (I) Dot chart of final tumor weight (n=5). (J) Dot chart of final tumor volume (n=5). (*p<0.05; **p<0.01; ***p<0.001 when compared with the vector group).

To study the tumorigenic ability of *EXO1 in vitro* and *in vivo*, western blot was used to detect the expression level of *EXO1* in 10 different HCC cell lines and one immortal liver cell line, HL-7702 (Figure 3A). The results showed that *EXO1* was upregulated in HLF and Huh7 cell lines, but downregulated in the Bel-7402 cell line. Hence, three short hairpin RNAs (sh*EXO1*-788, sh*EXO1*-789, and sh*EXO1*-331) specifically targeting *EXO1* were stably transfected into HLF and Huh7 cell lines, and an empty vector was transfected into each cell line (abbreviate as vector) and used as negative controls. The *EXO1*-shRNA significantly reduced* EXO1* protein expression in HLF and Huh7 cells compared with their control vectors in the western blotting results and this result was also verified by RT-PCR (Figure 3B). *EXO1* was also stably transduced into Bel-7402 cell lines and an empty vector was used as a control. Western blot and RT-PCR verified that *EXO1* was significantly overexpressed in transfected Bel-7402 cells (Figure 3C). Using functional assays, the tumorigenicity of *EXO1* was investigated. The Cell counting kit-8 (CCK-8) assay showed that* EXO1* knockdown in HLF and Huh7 cells significantly inhibited cell proliferation, while *EXO1* overexpression in Bel-7402 cells markedly promoted cell proliferation compared with their control vectors (*p*<0.01, Figure 3D, E). In the colony formation assay, the colony-forming ability of *EXO1*-knockdown cells was markedly lower than that of their control vectors (*p*<0.01, Figure 3F), whereas *EXO1*-overexpression in the cells showed significantly higher colony-forming ability than in the control vectors (*p*<0.01, Figure 3G). For *in vivo* tumorigenicity assays, HLF vector (control) and HLF-sh*EXO1*-788-transfected HCC cells were injected subcutaneously into nude mice (Figure 3H). As expected, the final tumor weights and volumes of the HLF-sh*EXO1*-788 cell-injected group were significantly reduced compared to that of the HLF vector cell-injected group (*p*=0.03, Figure 3I, J). These results indicate that *EXO1* has a strong promotive effect on cell proliferation and colony formation in HCC cells, and even promotes HCC tumorigenicity.

### *EXO1* promotes tumor invasion and metastasis *in vitro*

**Figure 4:**
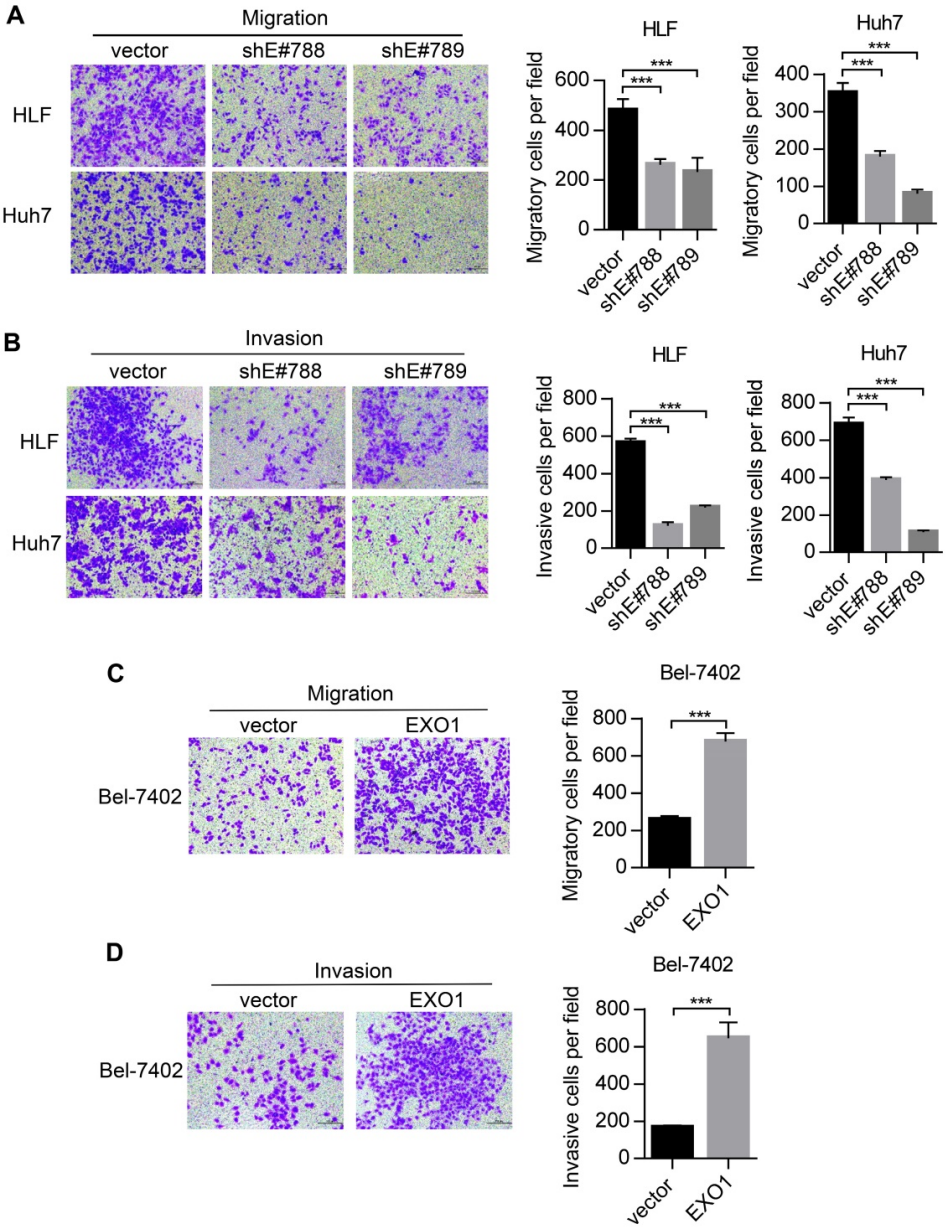
*EXO1* promotes migration and invasion of HCC cells *in vitro*. (A), (B) Representative images of migration and invasion of HLF and Huh7 cells by transwell migration and matrigel invasion assays. Bar chart shows that migration and invasion abilities were significantly reduced when *EXO1* was downregulated compared to the vector groups. The data are shown as the mean ± SD (n=6). Scale bar: 200 µm. (B), (C) Representative images of migration and invasion of Bel-7402 cells by transwell migration and matrigel invasion assays. The bar chart shows the migration and invasion abilities were significantly increased when *EXO1* was overexpressed compared to the vector groups. The data are shown as the mean ± SD (n=6). Scale bar: 200 µm (***p<0.001 when compared to the vector group).

Metastasis is one of the reasons for poor outcomes in HCC. To further explore whether *EXO1* can affect the invasion and metastatic abilities of HCC cells, we performed transwell assays. As shown in Figure 4A, migratory abilities were markedly decreased when *EXO1* expression was downregulated by shRNA in HLF and Huh7 cells compared to their respective controls. Additionally, as shown in Figure 4B, invasive abilities of HLF-sh*EXO1* and Huh7-sh*EXO1* were observably reduced. In contrast, the migratory and invasive abilities of Bel7402-*EXO1* cells overexpressing *EXO1* were much higher than their corresponding controls (Figure 4C, D). Altogether, these results demonstrate that *EXO1* plays an important role in the invasion and metastasis of HCC.

### Prediction of upstream regulators of *EXO1* via bioinformatics analysis

**Figure 5:**
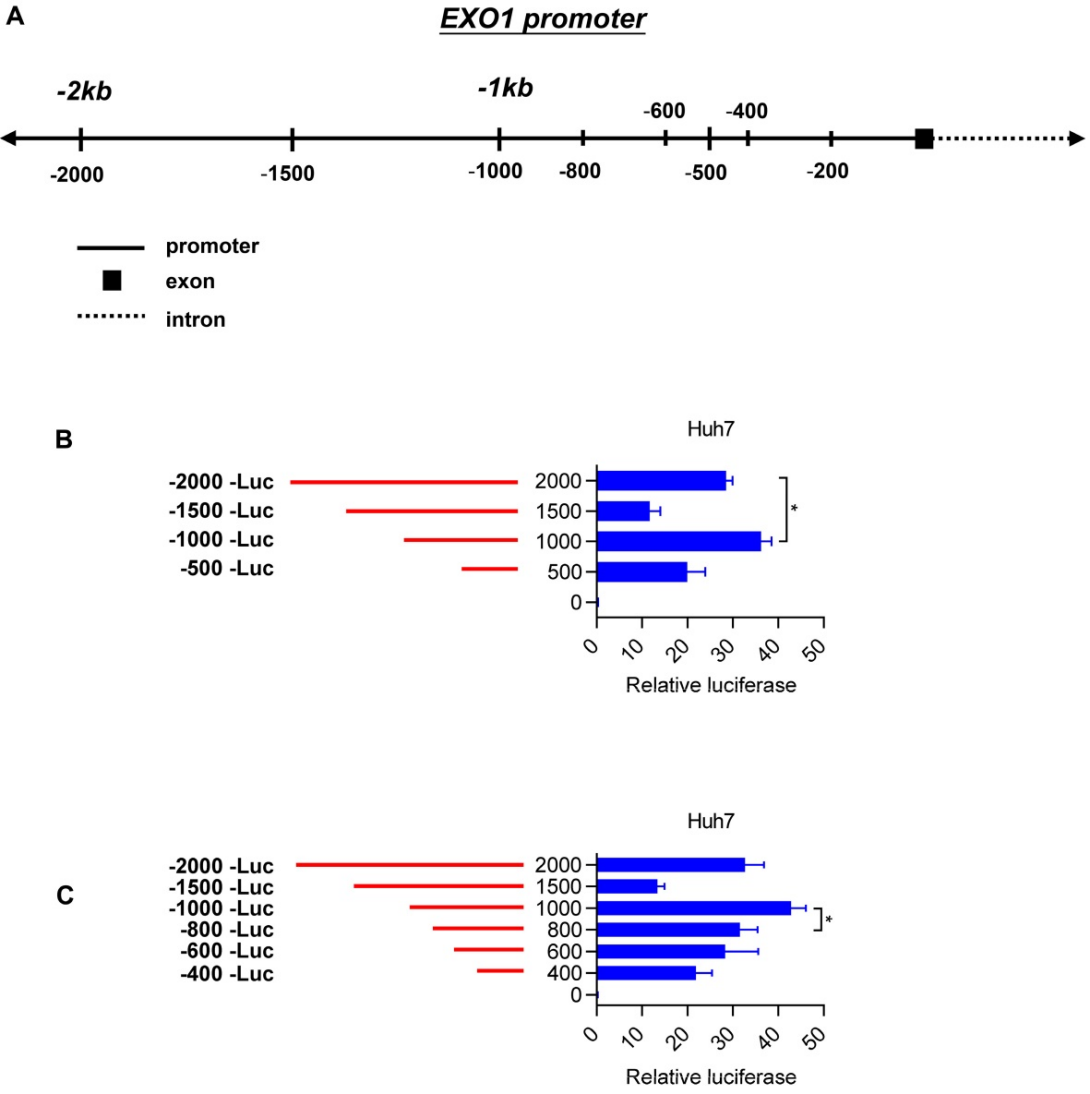
Truncation of *EXO1* promoter region. (A) Schematic diagram of the region lying 2000 bp upstream of *EXO1* transcription start site and a series of truncations. (B) Schematic diagram of the serial 500- promoter truncations (red) placed upstream of the *EXO1* translational start site and their corresponding luciferase reporter activities. Data are represented as the mean ± SD (n=3). (C) Schematic diagram of the serial 200- promoter truncations (red) placed upstream of the *EXO1* translational start site and their corresponding luciferase reporter activity. Data are represented as the mean ± SD (*n*=3). (*p<0.05).

To elucidate the molecular mechanisms of *EXO1* overexpression in HCC tumor tissues, we concentrated on the region 2 kb upstream of the transcription start site (TSS) of *EXO1* (Figure 5A). Introduction of this 2-kb region upstream of firefly luciferase in pGL4.17-basic yielded significantly greater reporter activity compared with the promoter-less construct in Huh7 cell lines (28-fold) (Figure 5B). With serial truncations of the 2 kb sequence at 500-bp intervals, we generated a set of luciferase reporter constructs (500 bp, 1000 bp, 1500 bp, 2000 bp). The luciferase reporter assay also showed a remarkable rise in luciferase activity when the upstream sequence of TSS of *EXO1* was 1000 bp long, and its luciferase activity was the strongest of the four sequences (*p*<0.05, Figure 5B). To further determine whether there is a region with higher transcriptional activity involved in the 1000-bp sequence, we continued to generate luciferase reporter constructs with truncations of the 1000-bp sequence at 200-bp intervals, which were 200 bp, 400 bp, 600 bp, and 800 bp upstream of the TSS of *EXO1*. As shown in Figure 5C, the relative reporter activity of the 1000-bp sequence was still the highest compared to other sequences. These results suggest that the 1000-bp region directly upstream of the TSS of *EXO1* possesses the highest transcriptional activity, and transcription factors possibly bind to the 1000-bp segment of *EXO1* promoter.

### *FOXP3* could influence the expression of *EXO1* in HCC

**Figure 6:**
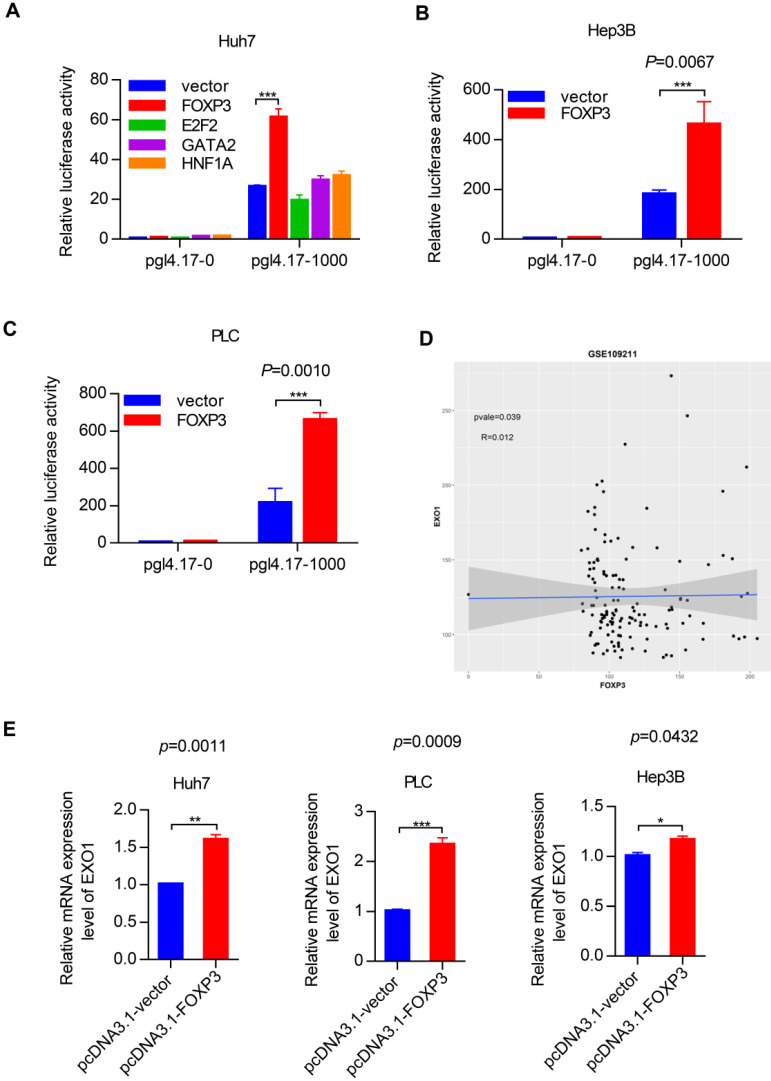
*FOXP3* enhances the expression of *EXO1*. (A) Bar chart of luciferase activity of transcription factors possibly binding to *EXO1* promoter region compared to the pcDNA3.1-vector group. Data are represented as the mean ± SD (n=3). (B), (C) pcDNA3.1 and pGL4.17 were co-transfected into Hep3B and PLC cells for 48 h. The bar chart of luciferase activity shows that when co-transfected with pcDNA3.1-*FOXP3* and pGL4.17-1000, the relative reporter activity was strongest. Data are represented as mean ± SD (n=3). (D) The scatterplot diagram of positive correlation between *EXO1* and *FOXP3* expression level in GSE109211 (n=140). (E) pcDNA3.1-*FOXP3* or pcDNA3.1-vector were transiently transfected into Huh7, PLC, and Hep3B cells for 48 h. The expression of *EXO1* was examined by RT-PCR. Data are represented as mean ± SD (n=3). (*p<0.05; **p<0.01; ***p<0.001).

To identify the transcription factors that could bind to the promoter region of *EXO1*, we used the PROMO and JASPA websites to make predictions. According to the scoring system provided by the website and gene function itself, we focused on four transcription factors named *FOXP3*, *E2F2*, *GATA2* and* HNF1A*. Plasmids were constructed with four coding sequences of these transcription factors mentioned above using pcDNA3.1 as a vector. We detected the transcriptional activity of these four transcription factors in the Huh7 cell line by luciferase reporter assay. Interestingly, as Figure 6A shows, *FOXP3* had a significantly high reporter activity level compared to the other transcription factors. Based on this crucial result, we performed the same luciferase reporter assay in the Hep3B and PLC cell lines. As shown in Figure 6B and 6C, compared with co-transfection of the pcDNA 3.1 vector and pGL4.17-1000, when co-transfected with pcDNA 3.1-*FOXP3* and PGL 4.17-1000, there was strong reporter activity. These results strongly suggest that *FOXP3* may be involved in the transcriptional regulation of the *EXO1* gene. Through bioinformatics analysis in the GEO database GSE109211, we found that the expression of *FOXP3* and *EXO1* had a positive correlation in HCC (Figure 6D). Hence, we transiently transfected the pcDNA3.1-*FOXP3* plasmid into the three different HCC cell lines (Huh7, PLC, Hep3B) and transfected a pcDNA3.1 vector as a control. Consistently, the mRNA level of *EXO1* increased with *FOXP3* transfection (Figure 6E), as detected by RT-PCR. Together, these results show that *FOXP3* promotes the expression of *EXO1* mRNA levels and may regulate the expression of *EXO1*.

### Depletion of *FOXP3* enhances the proliferation effects of *EXO1 in vitro*

**Figure 7:**
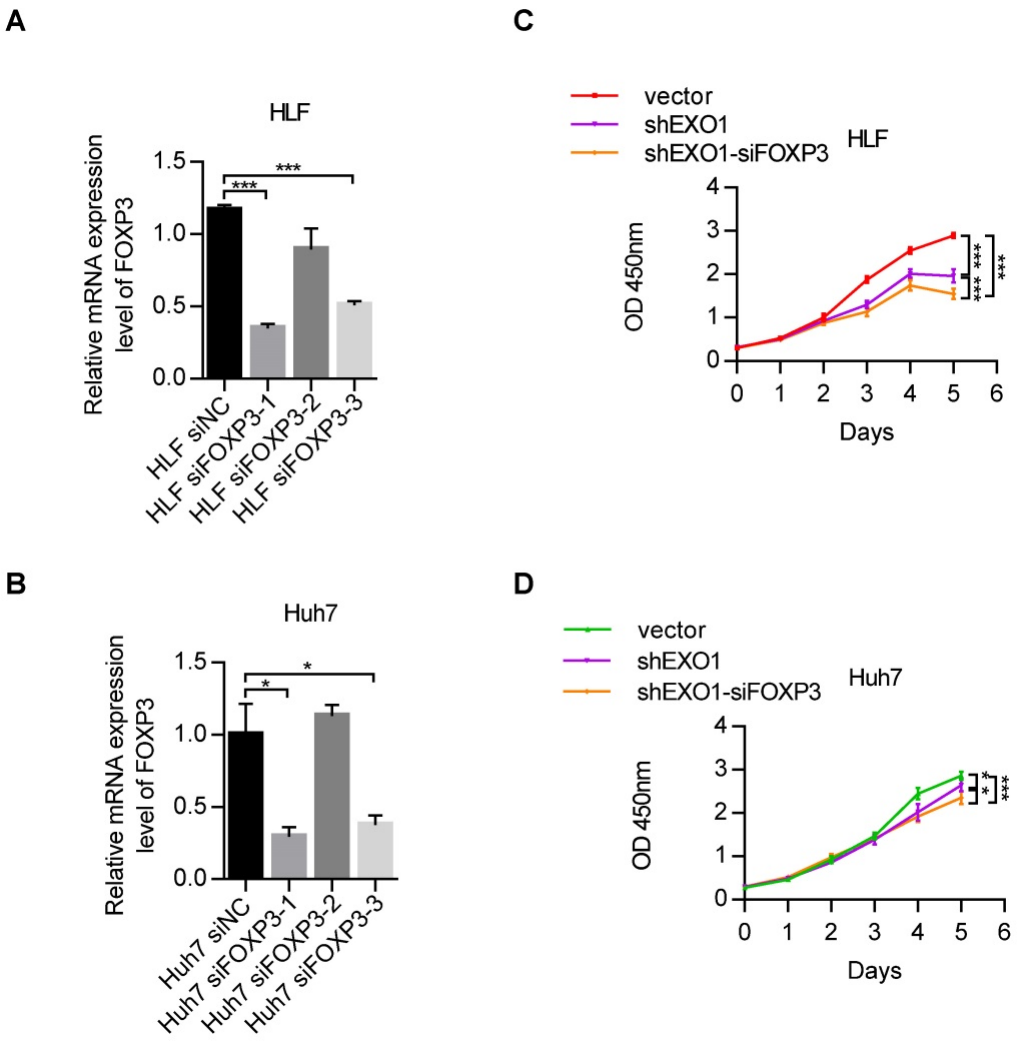
Depletion of *FOXP3* enhances the proliferative effects of *EXO1 in vitro* (A), (B) RT-PCR confirmed that *FOXP3* expression was effectively repressed by si*FOXP3*-1 in HLF and Huh7 cell lines. The data are shown as the mean ± SEM. (C), (D) CCK-8 assays indicated that knockdown of *FOXP3* in *EXO1* knockdown cells significantly reduced cell proliferation abilities compared to the *EXO1* knockdown alone. The data are shown as the mean ± SD (n=6). (*p<0.05; **p<0.01; ***p<0.001).

Due to the regulatory impact of *FOXP3* on *EXO1*, we wanted to explore whether *FOXP3* can affect the proliferation activity of *EXO1* in HCC cells. RT-PCR showed that among the three si*FOXP3* (si*FOXP3*-1, si*FOXP3*-2, and si*FOXP3*-3) knockdown sequences, *FOXP3* was significantly downregulated by si*FOXP3*-1 in HLF and Huh7 cell lines (*p*<0.05, Figure 7A, B). Next, we transfected si*FOXP3*-NC into sh*EXO1*-vector HCC cells and sh*EXO1*-788 HCC cells, respectively, and transfected si*FOXP3*-1 into sh*EXO1*-788 HCC cells. As shown in Figure 7C and D, CCK-8 assays indicated that compared with HCC cells with only *EXO1* knockdown, *FOXP3* and *EXO1* double knockdown significantly decreased the proliferation ability of the HCC cells (*p*<0.05). These results suggest that depletion of *FOXP3* further reduces cell proliferation ability after knocking down of *EXO1 in vitro*, suggesting that *FOXP3* may upregulate *EXO1*, and depletion of *FOXP3* may reduce its expression.

## Discussion

It has been shown that DNA damage caused by the hepatitis B virus (HBV) infection is one of the main molecular mechanisms of hepatocellular carcinoma 35-37. HBV-DNA can be integrated into host genomic DNA and cause insertion mutations or other genomic instabilities 38, 39. Studies have shown that DNA double strand breaks (DSBs) induced by DNA damage are potential targets for HBV-DNA integration 40-42. The body repairs DSBs through two major repair pathways: homologous recombination (HR) and non-homologous end-joining (NHEJ) 43. Bill et al. previously suggested that integration of duck hepatitis B virus (DHBV) DNA into the host hepatocyte genome may be accomplished by the body's NHEJ repair of DSBs, and the integration could occur at the sites of DNA damage 44. *EXO1* plays an important role in DNA damage repair, especially in HR and NHEJ after DSBs 45-48. As HR and NHEJ may participate in the integration of HBV-DNA, the relationship between *EXO1* and HBV related HCC is worth investigating. In the present study we observed that *EXO1* was frequently overexpressed in HCC samples and the same result was also seen in the Oncomine database. Analysis of *EXO1* prognosis from the TCGA database showed the overall survival rate and tumor-free survival rate of patients with high *EXO1* expression were significantly lower than those of patients with low *EXO1* expression. Thus, we concluded that *EXO1* is highly expressed in HCC and is associated with poor prognosis.

The link between *EXO1* and cancer is still under continuous research and much progress has been made. As mentioned earlier, several SNPs in the *EXO1* gene are related to the occurrence of certain tumors 17-25. Some *EXO1*-specific mutations such as E109K and A153V are thought to be located in the region required for nuclease activity, thereby deactivating protein function and affecting cancer susceptibility 49, 50. In the mouse model, the incidence of lymphoma in *EXO1*^null/null^ increased and the survival rate decreased, but the microsatellite instability (MSI) was not affected 13. *EXO1* has also been reported to be overexpressed in several other cancers as described in the introduction 15, 16, 51-53. In general, *EXO1* is poorly expressed and increased levels of *EXO1* may cause cellular damage due to its 5'-3' exonuclease activity, which may be contrary to previous studies 8, 54. This may be due to the complex role of *EXO1* in DNA damage repair and its tissue-specific expression. In humans, *EXO1* is highly expressed in the testis and moderately expressed in the thymus, colon, and placenta 5, 55. The liver has high mitochondrial activity and increased reactive oxygen species (ROS), which may lead to increased activation of the DNA damage repair pathway, resulting in a relatively high expression level of *EXO1* in the liver 55. Muthuswami et al. treated breast cancer cell line, MCF7, with different alkylating agents such as carboplatin, cyclophosphamide, etc. to induce DNA repair and found that *EXO1* expression increased with increasing concentration of these alkylating agents 51. de Sousa et al. observed that *EXO1* knockdown supported a faster DNA-DSB restoration after irradiation (IR) exposure in glioblastoma cell line T98G 15. All these, explain to some extent that *EXO1* is involved in the DNA repair pathway under cancerous conditions. The overexpression of *EXO1* in tumors may also increase the genetic instability and contribute to tumor initiation and progression through its involvement in recombinant events such as DSBs and telomere stabilization 56.

At present, the research on the role of *EXO1* in HCC is still lacking. Previously, it was reported that SNPs K589E (rs1047840) and rs3754093 of *EXO1* could increase and decrease the susceptibility of HCC, respectively 17, 18. In 2018, Dai et al. used clonogenic assays to find that the expression of *EXO1* affects the HCC cell survival under irradiation, and *EXO1* overexpression has a poor prognosis for HCC patients 16. In other types of tumors, as previously mentioned, Muthuswami et al. and de Sousa et al. investigated the possible role of *EXO1* in different types of tumors through the breast cancer cell line MCF7 and the glioblastoma cell line T98G, respectively 15, 51. In 2019, Luo et al. found the differential expression of *EXO1* in five prostate cancer cell lines, and the *EXO1* expression in the other four high‐invasive/metastatic prostate cancer cell lines significantly increased compared with the low‐invasive/metastatic potential cell line LNCaP 53. These researches above led us to explore the role of *EXO1* in the malignant biological characteristics of HCC by using HCC cell lines *in vitro* and *in vivo*. In this study, we observed that knockdown of *EXO1* reduced cell proliferation *in vitro* and *in vivo*, while overexpression of *EXO1* enhanced it. Subsequently, transwell assays showed that *EXO1* could markedly increase the cell motility of HCC cells* in vitro*, indicating that *EXO1* can affect the migration and invasion of HCC cells. Altogether, these functional assays indicate that *EXO1* may play a carcinogenic role in HCC. However, previous research reported that knockdown of *EXO1* in mice exhibits a mild phenotype, which may be contrary to our experimental results 57. This may be due to complex environmental factors *in vivo* as well as the multiple roles the *EXO1* gene itself plays in DSBs. There are still some researches suggesting that the absence of *EXO1* in mice will show MMR defects, apoptotic defects, and sterility 10, 11. Therefore, although we suggest that *EXO1* may function as a tumorigenic gene *in vitro*, more *in vivo* experiments are needed to validate these conclusions.

*EXO1* has been previously reported to be regulated by a variety of molecules and to bind to certain specific proteins 58, 59. Studies on DNA mismatch repair (MMR) have reported that *EXO1* interacts with the mismatch repair factors mutL homolog 1 (MLH1), mutS homolog 2 (MSH2), mutS homolog 3 (MSH3), and proliferating cell nuclear antigen (PCNA) to excise the newly synthesized DNA sequences containing errors during the MMR process 60-63. Checkpoint regulatory proteins 14-3-3 are widely reported as interaction partners of *EXO1*. Engels et al. identified in 2011 that in yeast and mammalian cells, 14-3-3 proteins interact with *EXO1 in vivo* to regulate the phosphorylation status of *EXO1*, thereby promoting *EXO1*-dependent fork progression after inhibiting replication 64. In 2012, Andersen et al. demonstrated specific interactions between *EXO1* and all the 7 isoforms of 14-3-3 through *in vitro* GST pull-down assays, and they demonstrated that the binding may involve a second unrecognized binding motif in *EXO1*, the amino acid S746. They also demonstrated that the 14-3-3 association may affect the nuclease activity of *EXO1* 65. In 2015, Chen et al. showed that 14-3-3 can interact with the central portion of *EXO1*, at least partially, by suppressing its association with PCNA, to negatively regulate *EXO1* damage recruitment and thus prevent excessive DNA excision 66. However, very little is known about the transcriptional regulation of *EXO1*. Muthuswami et al. discovered that transcription factors *E2F* and *Myc* may be involved in the regulation of *EXO1* by using luciferase reporter assays 51. In this study, we uncovered a new potential transcriptional regulator of *EXO1*, *FOXP3*, by truncating the promoter region of *EXO1* and using bioinformatic prediction. *FOXP3* has been proposed to function as a tumor suppressor in breast and prostate epithelial cells 67, 68. However, *FOXP3* could also be a prognostic factor in breast cancer and play an important role in the progression of cervical cancer 69, 70. In a recent report from 2019, Ou et al. reported that *FOXP3* silencing may be associated with the inhibition of cell proliferation and migration of HCC cells, as well as the induction of apoptosis 71. By transiently transfecting *FOXP3* into HCC cell lines, we observed that *FOXP3* could promote the transcriptional activity of *EXO1* by a luciferase reporter assay. We further found that *EXO1* mRNA levels were significantly upregulated after transient transfection of *FOXP3*. Moreover, bioinformatics analysis revealed a positive correlation between the expression of *EXO1* and *FOXP3*, and a CCK-8 assay in HCC cells proved that *FOXP3* could enhance the proliferative effect of *EXO1 in vitro*. These results indicate that *FOXP3* could upregulate the transcription of *EXO1* and might further regulate the expression level of *EXO1*. This is consistent with the reported results of Ou et al. Further explorations are required to validate this perspective.

Nonetheless, this study has some limitations. Firstly, the size of the clinical samples in this study was relatively small, with only 99 cases. Therefore, the number of samples assessed needs to be expanded. Second, there were database limitations and the expression level of *EXO1* and the prognosis of HCC patients need to be further verified by complete clinical follow-up. Finally, it is not uncommon that DNA repair genes are highly overexpressed in HCC. In addition to the possible explanations given above, X-Ray Repair Cross Complementing 2 (*XRCC2*) has also been reported to be up-regulated in a variety of tumors 72, 73. It is possible that DNA damage and repair processes are more active in tumor cells. RAD51B, RAD51C, RAD51D, and *XRCC2* form the BCDX2 complex, which promotes the aggregation of RAD51 protein at HR sites 74. The mechanism of *EXO1* overexpression in HCC is complex and still needs to be further explored.

In summary, our research found that *EXO1* was overexpressed in HCC and played a carcinogenic role in HCC, and that *FOXP3* may upregulate the expression level of *EXO1*. For the first time, we have uncovered a possible transcriptional regulation mechanism of *EXO1*. This discovery provides clues for the relationship between DNA damage repair genes and HCC and might lead to the development of novel anti-cancer therapeutics for HCC treatment.

## Figures and Tables

**Table 1:** Expression of DNA damage repair molecules increased by more than 10-fold

GenBank accession	Gene name	Description	Fold change
NM_130398	*EXO1*	Exonuclease 1	27.2*
NM_005431	*XRCC2*	X-ray repair complementing defective repair in Chinese hamster cells 2	17.5
NM_001789	CDC25A	Cell division cycle 25 homolog A	15.8
NM_002875	RAD51	RAD51 homolog	13.8
NM_001274	CHEK1	CHK1 checkpoint homolog	13.6
NM_032043	BRIP1	BRCA1 interacting protein C-terminal helicase 1	11.4
NM_012238	SIRT1	Sirtuin 1	11.4
NM_001790	CDC25C	Cell division cycle 25 homolog C	10.8

**Table 2:** The correlation between *EXO1* expression and clinicopathological features in HCC

Clinical variables	No. of patients	*EXO1* expression level		*p* value
	n=99	Low (n=21)	High (n=78)	
**Age (years)**				0.118
<60	66	17(81.0%)	49(62.8%)	
≥60	33	4(19.0%)	29(37.2%)	
**Gender**				0.123
Male	87	21(100.0%)	66(84.6%)	
Female	12	0(0.0%)	12(15.4%)	
**HBsAg**				1.000
Positive	88	19(90.5%)	69(88.5%)	
Negative	11	2(9.5%)	9(11.5%)	
**AFP(ng/ml)**				0.911
<400	67	14(66.7%)	53(67.9%)	
≥400	32	7(21.9%)	25(78.1%)	
**Child-Pugh Class**				0.152
A	88	21(100.0%)	67(85.9%)	
B	11	0(0.0%)	11(14.1%)	
**Liver cirrhosis**				**0.005**
No	19	9(42.9%)	10(12.8%)	
Yes	80	12(57.1%)	68(87.2%)	
**Tumor size (cm)**				0.709
≤5	46	9(42.9%)	37(47.4%)	
>5	53	12(57.1%)	41(52.6%)	
**Tumor number**				0.589
Single	86	17(81.0%)	69(88.5%)	
Multiple	13	4(19.0%)	9(11.5%)	
**Tumor encapsulation**				0.227
Yes	63	11(52.4%)	52(66.7%)	
No	36	10(47.6%)	26(33.3%)	
**Vascular invasion**				0.094
Yes	22	8(38.1%)	14(17.9%)	
No	77	13(61.9%)	64(82.1%)	
**Tumor differentiation**				0.903
Well	12	2(9.5%)	10(12.8%)	
Middle	52	11(52.4%)	41(52.6%)	
Poor	35	8(38.1%)	27(34.6%)	
**TNM stage**				0.531
I-II	75	17(81.0%)	58(74.4%)	
III-IV	24	4(19.0%)	20(25.6%)	

## Abbreviations

*EXO1*: Exonuclease 1
*FOXP3*: Forkhead box P3
HCC: Hepatocellular carcinoma
TNM: Tumor-node-metastasis
AFP: α-fetoprotein
ALT: alanine aminotransferase
AST: aspartate aminotransferase
SD: Standard deviation
SEM: Standard error of mean
